# Depression, osteoporosis, serotonin and cell membrane viscosity between biology and philosophical anthropology

**DOI:** 10.1186/1744-859X-10-9

**Published:** 2011-03-30

**Authors:** Massimo Cocchi, Lucio Tonello, Fabio Gabrielli, Massimo Pregnolato

**Affiliations:** 1 Institute 'Paolo Sotgiu' Quantitative and Evolutionary Psychiatry and Cardiology, L.U.De.S. University, Lugano, Switzerland, Via dei Faggi. 4, Quartiere La Sguancia CH - 6912 Lugano Pazzallo, Switzerland; 2 Department of Medical Veterinary Sciences, University of Bologna, Bologna, Italy; 3 Quantumbiolab, Department of Drug Sciences, University of Pavia, Viale Taramelli, 2I, 27100 Pavia, Italy

## Abstract

Due to the relationship between biology and culture, we believe that depression, understood as a cultural and existential phenomenon, has clear markers in molecular biology. We begin from an existential analysis of depression constituting the human condition and then shift to analysis of biological data confirming, according to our judgment, its original (ontological) structure. In this way philosophy is involved at the anthropological level, in as much as it detects the underlying meanings of depression in the original biological-cultural horizon of human life. Considering the integration of knowledge it is the task of molecular biology to identify the aforementioned markers, to which the existential aspects of depression are linked to. In particular, recent works show the existence of a link between serotonin and osteoporosis as a result of a modified expression of the low-density lipoprotein receptor-related protein 5 gene. Moreover, it is believed that the hereditary or acquired involvement of tryptophan hydroxylase 2 (Tph2) or 5-hydroxytryptamine transporter (5-HTT) is responsible for the reduced concentration of serotonin in the central nervous system, causing depression and affective disorders. This work studies the depression-osteoporosis relationship, with the aim of focusing on depressive disorders that concern the quantitative dynamic of platelet membrane viscosity and interactome cytoskeleton modifications (in particular Tubulin and Gsα protein) as a possible condition of the involvement of the serotonin axis (gut, brain and platelet), not only in depression but also in connection with osteoporosis.

## Depression and existential analysis

Memory is not a place of filing and storage of data geographically placed in our brain, because the brain is not merely a 'bundle of neurons' vivisected in a laboratory. It is in fact the 'condition of possibility' of an integral being, of an organism having continuously interacting levels: from the most elementary *conatus sese conservandi *to the feeling of life [1].

The conscience, as individual expression, full of phenomena and meaning, originating from its biological roots, considers memory as the most authentic figure of life and death, or rather the original picture of tragedy that has always lived in us.

'Consider the cattle, grazing as they pass you by. They do not know what is meant by yesterday or today, they leap about, eat, rest, digest, leap about again, and so from morn till night and from day to day, fettered to the moment and its pleasure or displeasure, and thus neither melancholy nor bored. This is a hard sight for a man to see; for, though he thinks himself better than the animals because he is human, he cannot help envying them their happiness - what they have, a life neither bored nor painful, is precisely what he wants, yet he cannot have it because he refuses to be like an animal'.

This powerful consideration by Nietzsche, taken from one of his most meaningful works *On the Use and Abuse of History for Life *[2], recalls that tragedy is the element that marks unequivocally our life in the world, together with memory that obsessively reminds man how his openness to things is characterised by a completeness and a happiness that, as Jankélévítch said, occurs in the world as lightning event [3].

In short, happiness appears to us as a transitory event, almost like a furtive gift of the gods, where pain seems to live in us as a usual condition. Man is an ill animal, as much as he is 'open to the meaning' as projects embodied in the world, and, at the same time, he is inevitably struck by pain and, above all, by death, that is the implosion of every meaning.

Memory, shifting from the extended to the identity level, takes the shape of 'nostalgia for the centre'; that is, the lost Unit (that is to say the Principle from which humans originate) that from a cultural point of view can be found in the history of religions and in general in the human thought [4-7]. The 'nostalgia for the Centre' is mainly an erotic archetype, a tormenting desire of love and beauty, of ontological integrity and harmony, increasingly fuelled by the melancholy that in Schelling's view is a "veil that falls on everything", things whose finitude make us worried, anxious.

The question of ontological pain and the precariousness of life 'here and now' is absolutely crucial, because the 'cosmic silence' makes us dismayed in the width of spaces, as well as 'the silence of the other makes us feel alone in the small universe of relations' [8]. So question on the structural melancholy, paradigmatic expression of ontological pain, shouldn't be negletted due to its radical nature. Infact, even if often remains in the background, nearly frozen, exorcised, (see Pascal's *Divertissement*), it can never be cancelled, otherwise the deep meaning of existence will be lost. In this context, memory, through language, that is to say culture, makes us understand to what extent depression (ontological or structural, human pain that embodies the ontic data; that is, to say, the single historical periods and single biographies) is a middle ground between the memory of the lost original Principle or Centre merely symbolic or religious, and the unquenchable aspiration for re-integration.

In conclusion, after this existential recognition and in the light of the biological considerations on depression herewith developed, we formulated a study hypothesis that can be expressed as follows: since the biochemical characteristics of the depressed population are the same as those of the population suffering from scleroderma, and since all people suffering from scleroderma are depressed, but not *vice versa*, is it rational to think that, close to a cultural origin of depression (the question on human structural and ontological precariousness herewith examined), there is a structural biological origin too? In other words, is it rational to think that depression may be the possible cause of more serious pathologies (for example, is inflammatory bowel disease, or osteoporosis, or neuroinflammation a cause or an effect of depression)?

## Depression and biological considerations

**Figure 1:**
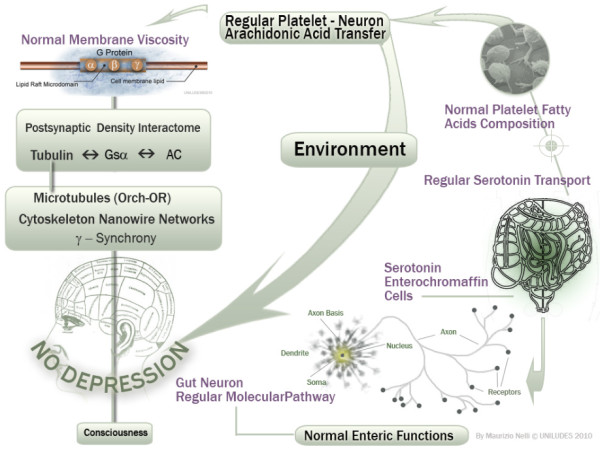
**Schematic description of the serotonin pathway from enterochromaffin cells (ECs) to platelets and interactome regulation through the membrane viscosity (depending from the regular arachidonic acid transfer from platelets to brain and *vice versa*) under normal conditions**.

**Figure 2:**
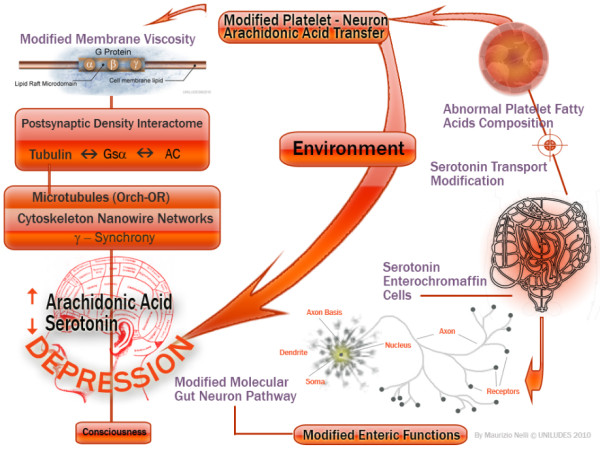
**Schematic hypothesis, in conditions of platelet membrane fatty acid modifications, of the serotonin pathway, from enterochromaffin cells (ECs) to platelets, and regulation of the interactome through the membrane viscosity**. When platelets reach a very high concentration of arachidonic acid, exchanges of arachidonic acid between platelets and the brain and *vice versa *are not possible; arachidonic acid increases in the brain and neurons, and platelet membranes become very fluid (loss of viscosity) impairing serotonin uptake in both cells.

Recently, from an experimental basis, the molecular depression hypothesis [9] and the involvement of interactome [10] have been formulated, as shown in Figures 1 and 2.

It is well known that other assumptions about depressive disorder have exhaustively considered the platelet membrane as a bridge to psychiatric illness as a result of changes in viscosity [11]. When membrane viscosity is very low, due to the increase of arachidonic acid [12-14], the capacity of platelet and neuronal serotonin receptors to capture serotonin (5-hydroxytryptamine) [15] is impaired.

Since we know that serotonin does not cross the blood-brain barrier, the mechanism described could explain the strong similarity of the low concentration of serotonin neurons and platelets in depression [16-19]. In these circumstances, and in the absence of an efficient reuptake mechanism, some serotonin remains free in the brain neuronal domain, in the enteric neuron domain and in the circulation.

This can be a strong critical element in the relationship (direct and indirect) that a defect or an excess of serotonin (as in depression and other psychiatric disorders) can have with serious diseases such as scleroderma [20], bowel inflammation [21,22], multiple sclerosis [23], coronary heart disease [24], or osteoporosis [25], where a high incidence of depressive disorder is documented.

## Serotonin-bone connection

Rosen [26], when discussing the connection between bone, brain and intestine, describes the serotonin cycle in a detailed and comprehensive way: two different types of enzymes, tryptophan hydroxylase (Tph)1 and Tph2 promote the synthesis of serotonin in enterochromaffin cells and brain, respectively; serotonin released in the gut in part stimulates peristalsis and in part enters the bloodstream, is transported to platelets via the 5-hydroxytryptamine transporter (5HTT), and is stored or released during the process of coagulation.

In the central nervous system, serotonin, produced through the action of Tph2, is released at neuronal synapses. Its reuptake is controlled by the action of 5HTT. Since serotonin does not cross the blood-brain barrier, all its activity in the brain is mediated by phenomena of synthesis, reuptake and binding to 5HTT receptors.

This leads us to believe that the only changes in Tph2 or in 5HTT activity, by altering levels of serotonin in the brain, are the primary cause for the induction of osteoporotic disease [26]. This mechanism, moreover, would exclude the involvement of the platelets and their viscosity, as compared to the skeleton in its integration with brain and gut.

Analysis of Rosen [26] suggests that, as a result of lipoprotein receptor-related protein 5 (Lrp5) gene function loss, there are higher circulating levels of serotonin and that a deep modification of the 5HTT activity, adversely affects osteogenesis.

The results reported in the literature with regard to the involvement in osteoporosis of brain or intestinal serotonin, as well as its action on osteoclasts and osteoblasts, are conflicting. Rosen [26], citing Yadav's work [27], reports that intestinal serotonin inhibits bone formation independently from the activity of brain serotonin. In subsequent work, Yadav [28] reports that the influence of brain serotonin on bone mass takes precedence over that exerted by serotonin of intestinal origin. However, the same work emphasises how, according to the place of synthesis, serotonin regulates bone mass in different ways, inhibiting (duodenal serotonin) and favouring (brain serotonin) by attributing such effects to the serotonin condition of hormone or neurotransmitter.

The *New England Journal of Medicine *published a discussion about the article by Rosen [26]. Some authors disagree with Rosen's theory on the influence of serotonin, together with its origin and mode of transport, in the determination of the osteoporotic process.

Anderson *et al. *[29] stress the paradox that the increase in platelet serotonin in Lrp5 gene knockout rats and in subjects with osteoporosis pseudoglioma leads to bone loss, whereas treatment with selective serotonin reuptake inhibitors (SSRIs), which reduce platelet serotonin by about 80% to 95% is also responsible for the reduction of bone mass.

If serotonin apparently inhibits bone formation, it is puzzling that carcinoid syndrome is not commonly associated with osteoporosis.

de Jong *et al. *[30] emphasise that, after treatment with SSRIs, free serotonin should be high (it seems that the level of free serotonin after SSRI treatment is not known), making this condition similar to serotonin-producing subjects with metastatic carcinoid tumours, with the caveat that these subjects do not have any particular tendency to osteoporosis.

de Jong *et al. *[30] provide a possible explanation for the discrepancy, with regard to the metabolic clearance of serotonin.

Since SSRIs also reduce serotonin clearance in peripheral transporter-expressing target organs, such as bone, serotonin receptor activation is increased. In contrast, in patients with carcinoid tumours, transporter function is intact and metabolic clearance can be highly upregulated [31].

Rosen [32] makes observations stressing two main aspects of the mechanisms that cause the osteoporotic phenomenon. Firstly, with respect to the mechanism of bone loss from sympathetic activity, activated adrenergic receptors on osteoblasts suppress critical transcription factors necessary for bone formation but also enhance osteoclastogenesis, principally by upregulating the osteoclast differentiation factor receptor activator of nuclear factor κB ligand (RANKL) [33]. This is not a diversion of osteoblasts to osteoclasts, as noted by Speth [34], but rather a dynamic process of coupling that involves two cell types originating from distinct progenitor cells. Secondly, the mechanism of bone loss induced by SSRIs. [27]. Hence, it is conceivable that there is a balance in bone turnover between the central blockade of serotonin reuptake and changes that may be associated with circulating serotonin and its release from platelets.

Karsenty [35] states that inhibition of the serotonin of intestinal origin is an effective solution in the treatment of osteoporosis, and Battaglino *et al. *[36] state that experimental data suggest that serotonin plays a key role in bone homeostasis through an effect on osteoclasts differentiation. Regardless, there is unanimity of views, from the same authors, on the role of SSRIs in the osteoporotic process.

## Depression and osteoporosis

In this maze of conflicting evidences, on the basis of the strong probability that circulating serotonin can be invoked in osteoporosis and, further, for the possible liability of the SSRIs in the induction of the osteoporotic phenomenon, we consider the hypothesis that derives from research conducted on the relationship between membrane viscosity and serotonin plausible with regard to the possible role of osteoporosis in depression [10-15]. Awareness of a strong link between depression and osteoporosis [25] is growing, although a clear definition of the connection between the two diseases is not yet available.

**Figure 3:**
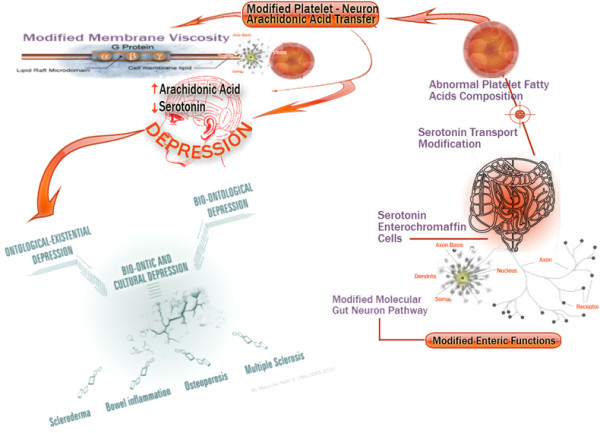
**The possible links between depression and other pathologies where serotonin is involved, according to the hypothesis of compromised serotonin transport**. The compromised serotonin transport, which depends on membrane viscosity, can cause conditions of increased or decreased serotonin uptake by platelets leading to altered platelet function that in turn could be involved in different pathologies.

On the basis of the research conducted on the role of platelets and their membrane fatty acids [37] it has been shown that the viscosity could be a focus of attention in order to allow a new interpretation of the serotonin receptor uptake [11,12] and of the relationship between depression, osteoporosis, and other diseases in which serotonin is involved with respect to possible anomalies in cell (platelet and neuron) concentration (Figure 3).

The above reasons make plausible, from a different point of view, that the involvement of depressive disorders on the osteoporotic phenomenon, as if to mimic SSRIs activity in their phase of initial platelet receptors reuptake inhibition, can cause a significant reduction of serotonin (see Figures 1 and 2).

In the sequence of the molecular events that can lead to fluctuation in the viscosity of the platelet membrane, namely the phenomenon of exchange of arachidonic acid from platelets and brain and *vice versa*, we have shown that when platelets are saturated with arachidonic acid this exchange is no longer possible [13,14] and the two areas of serotonin receptors (platelets and neurons) could have limitations of serotonin reuptake for the reduction of viscosity [15], freeing serotonin.

An excess of serotonin, as suggested by Rosen and comparing the two theories, probably complementary, may lead to an insult to the bone.

The activity of SSRIs on the one hand, leads to a reduction of depressive disorder by promoting the entry of larger amounts of serotonin in the brain on the other hand, continuing the inhibiting effect of the serotonin uptake on the platelet membrane receptors [38]. This, essentially leaves the serotonin decoupled from platelet receptors and could maintain the osteoporotic effect. In short, even during depression treatment, platelet membrane viscosity does not change, and it would remain a permanent stimulus to the impairment of bone homeostasis and of other diseases that have serotonin inbalance as a common feature.

## Conclusions

At the time it is shown that the viscosity of the platelet membrane is a general influencing factor for serotonin receptor uptake, a general principle governing the handling of serotonin itself is established with regard to its relations with the depressive disorder. It may also be involved, to a certain extent, in some pathologies that recognise serotonin changes; that is, scleroderma, inflammatory bowel disease, neuroinflammation, multiple sclerosis and osteoporosis.

The high incidence of depression, in these pathological conditions, leads us to consider a general phenomenological rule rather than a specific error in gene expression or loss of enzyme function. The viscosity of the membrane appears to be a concept more plausible than the fact that it is a phenomenon to which changes may contribute more general factors compared to the exclusivity of a gene expression error and/or an abnormality of enzyme function. The serotonin receptors and their subtypes, together with the modification of gene expression of transporters, represent a very complex and intriguing network. We must understand if it is possible to find a general and common rule to explain all the different molecular aspects of the serotonin pathways, tissue connections and responsibilities in its involvement in pathologies.

The platelet molecular error identified in depression [9,11] seems to not be irreversible in all subjects, but could, in some cases, be partially recoverable by correction of membrane viscosity.

This refers to the portion of the population that may experience mood disorders of varying intensity where there is defective membrane dependent transport of serotonin, which could also affect correct osteblastogenesis.

The concept of membrane viscosity [11] and the implications that are reflected on the molecular homeostasis of the cytoskeleton, can be the link to which future research to better understand the complex phenomenon should be directed at, which bidirectionally links the brain-gut axis in relation to serotonin transport, and that likely makes the depressive condition the disorder to which other diseases may be related.

In depression, the evidence of a reduced uptake of serotonin in platelets and neurons, justifies the possible influence of free serotonin on bone mass regardless of origin, brain or blood.

**Figure 4:**
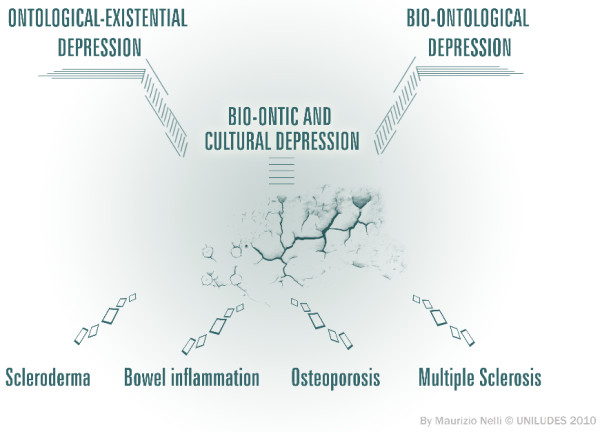
**In our interpretative model, depression stands out as the primary cultural and biological structure of living (ontological version) with all its pathology classifying and historical-biographical relapses (ontic version)**.

In conclusion, depression, is a logical correspondence between biological markers and biolocial-cultural expressions, considered as the essential interpretation key of human existence, from the biological point of view, as well as from a symbolic-cultural and existential perspective, according to Figure 4.

## Competing interests

The authors declare that they have no competing interests.

## Authors' contributions

All the authors made substantial contributions to the design and concept of the study. All the authors were involved in drafting and revising the manuscript and have read and approved the final manuscript.
